# Intergeneration Transmission of Violence in Forensic Patients With a Diagnosis of Schizophrenia and Psychosis: Was Parental Alcoholic Abuse a Significant Factor?

**DOI:** 10.3389/fpsyt.2021.765279

**Published:** 2021-12-02

**Authors:** Milena Petrovic, Lidija Injac Stevovic

**Affiliations:** ^1^Medical Faculty University of Montenegro, Podgorica, Montenegro; ^2^Clinical Center of Montenegro, Psychiatric Clinic, Podgorica, Montenegro

**Keywords:** traumatic events in childhood, forensic psychiatry, development psychopathology, psychosis, schizophrenia, intergenerational transmission of trauma

## Abstract

**Background:** Child abuse during childhood and the presence of parental alcohol abuse increase the risk of developing mental illness in children, as well as the risk of violent behavior in adulthood. The association of these factors has not been sufficiently investigated when it comes to forensic mental patients. In this study, we examined the impact of traumatic events in childhood and the presence of mental illness and alcohol abuse of parents in subjects with psychosis and schizophrenia who committed serious crimes.

**Methods:** One-hundred and forty-three respondents were included in the current study. Information on childhood abuse was collected by Childhood Trauma Questionnaire (CTQ). The sample included participants diagnosed with psychotic disorders and schizophrenia with a history of violent behavior (PSCH-V, *n* = 20), patients diagnosed with psychotic disorders and schizophrenia without a history of violent behavior (Non-V-PSCH, *n* = 51), and healthy control patients (HC, *n* = 72). Participants were diagnosed according to the ICD 10 classification system. MINI and CAINS scales were used to confirm the diagnosis. Data on sociodemographic and clinical characteristics were collected. Differences between groups in terms of traumatic events in childhood as well as parental alcohol abuse are presented and analyzed, using descriptive statistical values and nonparametric techniques of inferential statistics.

**Results:** Statistically significant differences were obtained for total scores (χ^2^ = 28.522, *p* < 0.001) as well as for (1) major upheaval between the parents (χ^2^ = 20.739, *p* < 0.001), (2) being victim of violence—other than sexual (χ^2^ = 12.701, *p* < 0.01), and (3) other major upheaval that may have shaped life or personality significantly (χ^2^ = 30.920, *p* < 0.001). PSCH-V, compared to HC, had greater exposure to all of the three domains of childhood trauma (*U* = 396.500, 436.500, and 376.000, respectively; *p* < 0.001). Similar results were obtained when Non V-PSCH were compared with HC (*U* = 1,223.000, 1,535.000, and 999.000, respectively; *p* < 0.001). The results indicated statistically insignificant differences between PSCH-V and Non-V-PSCH in having a family history of mental illness. On the other hand, family history of mental illness was less present in HC compared to PSCH-V (χ^2^ = 24.238, *p* < 0.001) and Non V-PSCH (χ^2^ = 14.456, *p* < 0.001). The presence of parental alcohol abuse was predominantly present in the PSCH-V group (60%) while a significantly lower presence was found in the Non-V PSCH group (35%) and HC (5.5%).

**Conclusion:** Both PSCH-V and Non-V-PSCH groups had a high degree of exposure to traumatic events in childhood compared to the HC. In PSCH-V, the presence of parental alcohol abuse compared to Non-V-PSCH was dominant. Mental illness coupled with a history of violent behavior represents a factor of polyvictimization, which may increase the likelihood of violent behavior of offspring.

## Introduction

Schizophrenia and other psychotic disorders are considered an important risk factor for violence, since schizophrenia and psychosis patients are at small but significantly increased risk in engaging in violent behavior. So far, the exact mechanism of how to predict whether a person being treated for psychosis will exhibit violent behavior is not known. Research has shown that violent behavior is often associated with psychotic symptoms, decreased impulse control and substance abuse, but also traumatic childhood events such as physical, sexual, and emotional abuse (1). There is a lack of research of violent behavior in psychiatric forensic patients (2).

Research has shown that individuals who have been exposed to traumatic childhood events, such as violence and abuse, are at increased risk of exhibiting violent behavior later in life. This phenomenon has been regarded as intergenerational transmission of violence. Children who have suffered childhood abuse have an increased risk of developing a variety of mental problems, including personality disorder, psychosis, schizophrenia, as well as substance abuse, which has been predominantly present among forensic mental health inpatients (3, 4). It has been found that, when compared to the general population, people with severe mental disorders, who were exposed to the most severe forms of childhood abuse, are at an increased risk of later violent behavior toward other people (5, 6). Bruce and Laporte reported that hospitalized forensic patients diagnosed with schizophrenia and schizoaffective disorder who were victims of childhood physical violence were 170% more likely to engage in violent behavior within their community before admission to the hospital (7). Also, studies that investigated different types of childhood abuse have shown the phenomenon of polyvictimization, that is, that there has been an association of multiple forms of abuse (2). Previous studies of child abuse according to the CTQ scale have shown a significant percentage of those who have had a high degree of childhood trauma in general among patients with psychotic disorders. The most significant variable was physical violence.

Children raised in families whose parents were abusing alcohol are especially vulnerable. Parents who are abusing alcohol have a reduced ability to provide a safe environment for their children and to respond adequately to the children's physical and emotional needs. This may further lead to different adverse consequences for children. Children whose parents are abusing alcohol have more frequent cognitive and emotional problems (8, 9). Additionally, they more often experience behavioral problems and have a tendency to develop a mental disorder later in adolescence and adulthood (10). Parental alcohol abuse is also associated with other problems in the family, such as lack of education and the appearance of mental problems (11), which can further complicate the lives of children. These children are often exposed to physical abuse, they witness the violent behavior of their parents toward other family members, there are more frequent quarrels between the parents, and in some cases separation. Early and long-term risks to children's psychosocial health are very important factors leading to inequalities in the social sphere (12). Studies have shown that there is a lack of research focused on children of parents who are abusing alcohol and their needs (12, 13).

Professionals who provide services to parents who are abusing alcohol are not sufficiently trained and instructed to work with their children. Although there are services that should be able to provide support to children from these families, they face a lack of professionals such as selected doctors, teachers, social workers, psychologists, especially those who are trained in this area. Early interventions are an opportunity to provide quality support and follow-up to prevent a possible escalation of a mental disorder (13).

The aim of the current study was to examine exposure to different types of childhood abuse and violent behavior, on a sample of patients with a diagnosis of psychosis and schizophrenia who committed crimes and patients with psychosis and schizophrenia who did not exhibit violent behavior in relation to the healthy control group. We examined group differences in terms of the level of overall childhood abuse score as well as all subdomains on the CTQ scale. We also examined the socio-demographic and clinical characteristics, among which the alcoholism of the parents stood out, which were spontaneously presented during the anamnesis by the patients who committed the crimes.

According to our hypothesis, violent patients with psychosis and schizophrenia were exposed to higher overall childhood traumas, including abuse and neglect, compared to patients with psychosis and schizophrenia who did not exhibit violent behavior, and healthy control group.

## Methodology

The sample included 143 male respondents.

The first group consisted of 20 patients with psychotic disorders and schizophrenia, who were convicted of criminal acts of violent behavior (murder, attempted murder, domestic violence, violent burglary, and kidnapping) and who were sentenced to compulsory treatment in hospital at the Forensic Department Specialized Psychiatric Hospital Dobrota in Kotor.

The second group consisted of 51 patients, who were hospitalized due to psychosis or schizophrenia and who do not have a history of violent behavior, and who are treated at the Specialized Psychiatric Hospital Dobrota in Kotor and the Clinic for Psychiatry of Clinical center of Montenegro, in Podgorica.

The third group of healthy control patients consisted of 72 voluntary blood donors.

Inclusion criteria were based on gender. Recruited respondents were 18–70 years old at the time of inclusion in the study. Patients were recruited in the period from 2019 to 2021. The study was approved by the Ethics Committee of the Medical Faculty and was conducted in two large psychiatric hospitals which provide mental health services to the largest number of patients from all over the country, Specialized Psychiatric Hospital Dobrota in Kotor and the Clinic for Psychiatry of Clinical center of Montenegro in Podgorica. This research is part of the doctoral dissertation “Investigation of the influence of polymorphisms in the COMT, DRD2, and APOE genes on the therapeutic response to antipsychotics in patients with psychotic disorders from the schizophrenia spectrum” whose development and implementation was approved by the Ministry of Education, Science, Culture, and Sports of Montenegro.

The study is being conducted in accordance with the Helsinki Declaration. All participants who voluntarily accepted to participate in the study were asked to sign an informed consent. Prior to that, they were informed about the purpose of the study.

The clinical assessment was performed by a resident in psychiatry and a psychiatrist. These professionals conducted the safety assessment during the interview with patients who exhibited violent behavior. From this group, four patients did not accept cooperation and therefore were not included in the study. From the second group of patients who did not exhibit violent behavior, three patients refused to participate in the study.

Patients were diagnosed according to the ICD 10 classification system, and the MINI and CAINS scales were used to confirm the diagnosis. Screening for the ability to give informed consent was conducted as well. MMSE and UCABB scales were used for the first two groups of participants.

The basic data and clinical characteristics of the respondents were collected by a socio-demographic questionnaire, designed for the needs of this study. This questionnaire provided data on age, marital status, level of education, employment, smoking habits, diagnosis according to ICD 10 classification system, number of hospitalizations, duration of illness, period of onset of illness, course of illness, family history of mental illness, and presence of alcoholism in the primary family.

To assess childhood abuse, the Childhood Trauma Questionnaire was used, a commonly used instrument with satisfactory psychometric characteristics, that assesses retrospectively traumatic childhood events. It consists of six subscales, which measure physical, emotional, sexual abuse, as well as the existence of illness in early childhood, loss of a family member, and divorce/separation of parents. The scale also estimates how much patients confided in others in relation to the traumatic events they experienced in childhood.

In order to calculate the level of exposure to childhood trauma, negative answers to questions from CTQ were coded as “0” and if the answer was “yes”, there was a 7-point Likert scale (1–7) to estimate the level of exposure. The minimum possible score was 0 and the maximum possible score was 7. Higher scores represent higher levels of exposure to childhood trauma, in other words, worse traumatic experiences during childhood. Total scores are calculated by summing up the scores on all six items. In line with that, total scores theoretically range from 0 to 42.

SPSS for Windows (version 21.0) was used for statistical analysis. Non-parametric statistics were used for data processing—chi-square test (to examine the existence of statistically significant differences between frequencies), Mann–Whitney *U*-test (to examine the statistical significance of differences between two groups of subjects), and Kruskal–Wallis test (to examine the statistical significance of differences between the three groups of respondents).

## Results

Sociodemographic characteristics (Table 1) showed that respondents in the PSCH-V group were on average younger than the Non-V-PSCH group respondents. Additionally, respondents in the HC group were younger than respondents in the other two groups. The largest number of patients in the PSCH-V group were married (18; 90%), while in the Non-V-PSCH group the largest number of respondents were not married (38; 74.5%). In the HC group, there was almost an equal number of married and unmarried respondents (35; 48.61%) and (34; 47.22%), respectively.

**Table 1:** Sociodemographic and clinical characteristics of the sample.

	**Offenders (PSCH-V)** **(*n* = 20)**	**Non-offenders (Non-V-PSCH)** **(*n* = 51)**	**Healthy controls (HC)** **(*N* = 72)**
**Age**			
Mean (standard deviation)	37.60 (12.10)	44.96 (12.18)	33.61 (9.14)
**Marital status**			
Single	2	38	34
Married	18	6	35
Widower	0	2	2
Divorced	0	5	1
**Level of education**			
Non-formal	0	1	0
Elementary school	3	6	0
High school	15	38	27
College or university degree	2	6	30
Master of Arts/Science	0	0	10
PhD	0	0	5
**Total years of schooling**			
Mean (standard deviation)	11.80 (1.94)	11.98 (2.44)	16.15 (3.24)
**Employment status**			
Unemployed	12	26	1
Pupil	0	0	2
Student	1	2	6
Employed	2	11	63
Retired	5	12	0
**Diagnosis (ICD-10)**			
F20	5	12	N/A
F22	1	5	N/A
F23	3	9	N/A
F25	1	5	N/A
F28	0	1	N/A
F29	10	18	N/A
F31	0	1	N/A
**Illness duration (in years)**			
Mean (standard deviation)	14.58 (9.10)	19.38 (11.22)	N/A
Median (range)	13 (1.5-33)	19 (1–42)	N/A
**Age at illness onset**			
Mean (standard deviation)	23.05 (8.31)	24.84 (8.64)	N/A
Median (range)	23 (13–48)	24 (12–52)	N/A
**Number of hospitalization**			
Mean (standard deviation)	5.90 (7.83)	5.47 (8.04)	N/A
Median (range)	3 (1–27)	2 (0-40)	N/A
**Course of the illness**			
Continuous	16	39	N/A
Episodic	4	12	N/A
**Family history of mental illness of parents**			
Yes	15	24	11
No	5	27	60
**Smoking status**			
Current smokers	18	39	26
Past smokers	0	3	10
Never smokers	2	9	36
**Family history of alcoholism of parents**			
Yes	12	18	4
No	8	33	68

The level of education was approximately the same in the PSCH-V and Non-V-PSCH groups, while respondents from the HC group had 5 years more of education, on average. The largest number of respondents from the PSCH-V group were unemployed (12; 60%), as in the Non-V-PSCH group (26; 50.98%), while in the HC group, the largest number of respondents were employed (63; 87.5%).

According to the ICD 10 classification system, the largest number of participants from the PSCH-V group had a diagnosis of psychosis, followed by schizoaffective psychosis. In the Non-V-PSCH group, 18 (35.29%) patients were diagnosed with psychosis and 12 (23.52%) had a diagnosis of schizophrenia. The duration of the disease was shorter in subjects from the PSCH-V group (14.58) compared to 19.38 in Non-V-PSCH. In the PSCH-V group, the disease first appeared at an average age of 23 years, and in the Non-V-PSCH group at an average age of 24 years. In terms of the number of hospitalizations, approximately the same number of hospitalizations was present in both the PSCH-V group (5.9) and the Non-V-PSCH (5.47). The disease had a continuous course in 16 (80%) patients in the PSCH-V group, and in 39 (76.47%) patients in the Non-V-PSCH group.

Differences between family history of mental illness were tested with chi-squared test with Yates' correction due to the fact that some frequencies in the table were small. The results indicated statistically insignificant differences between PSCH-V and Non-V-PSCH in having a family history of mental illness; however, the *p*-value could be considered as marginal (χ^2^ = 3.472, *p* = 0.062). On the other hand, family history of mental illness was less present in healthy controls, compared to offenders (χ^2^ = 24.238, *p* < 0.001) and non-offenders (χ^2^ = 14.456, *p* < 0.001).

In the PSCH-V group, we have identified 18 (90%), current smokers, while in the Non-V-PSCH this number was 39 (76.47%), which indicates a higher number compared to the HC group, where we have identified 26 (36.11%) current smokers.

The presence of parental alcohol abuse was predominantly found in the PSCH-V group [12 of 20 patients (60%)] while this number was significantly smaller in the Non-V-PSCH group [18 of 51 (35%)] and HC [4 of 72 subjects (5.5%)].

**Figure 1:**
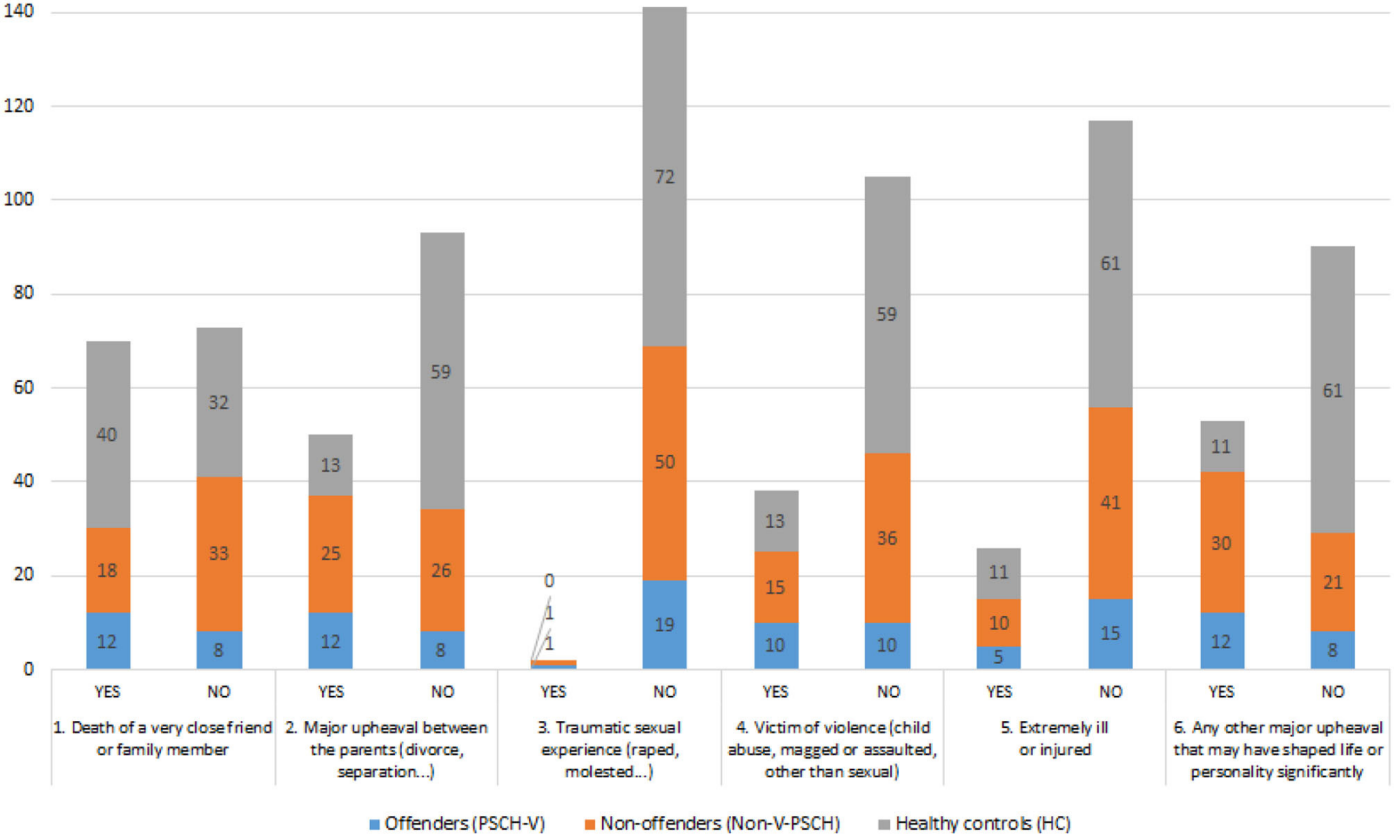
Frequencies of participants who have or have not experienced each domain of childhood trauma.

Descriptive values (frequencies) regarding the experiences of various domains of childhood trauma are shown in Figure 1.

Comparisons in exposure to childhood trauma between groups were done by using the Kruskal–Wallis test (Table 2). Higher mean ranks indicate higher exposure to different traumatic experiences during childhood. The rationale behind the decision to use the mentioned statistical test was the imbalance of subsample sizes as well as the fact that subsamples were small.

**Table 2:** Results of Kruskal–Wallis test for differences between offenders, non-offenders, and healthy controls in exposure to childhood trauma.

**Traumatic events and total exposure to childhood trauma**	**Mean ranks**			**χ^2^**	***p***
	**Offenders (PSCH-V)**	**Non-offenders (Non-V-PSCH)**	**Healthy controls (HC)**		
1. Death of a very close friend or family member	80.22	64.43	75.08	3.367	0.186
2. Major upheaval between the parents (divorce, separation...)	90.98	82.92	58.99	20.739	0.000
3. Traumatic sexual experience (raped, molested...)	74.58	72.40	71.00	2.998	0.223
4. Victim of violence (child abuse, magged or assaulted, other than sexual)	91.88	75.67	63.88	12.701	0.002
5. Extremely ill or injured	76.90	72.43	70.33	0.918	0.632
6. Any other major upheaval that may have shaped life or personality significantly	90.50	87.90	55.60	30.920	0.000
Total scores	103.50	83.89	54.83	28.522	0.000

As can be seen in Table 2, there were no statistically significant differences between the three groups in exposure to the following traumatic events: (1) death of a very close friend or family member, (2) traumatic sexual experience (raped, molested...), and (3) extremely ill or injured.

On the other hand, statistically significant differences were obtained for total scores (χ^2^ = 28.522, *p* < 0.001) as well as for (1) major upheaval between the parents (χ^2^ = 20.739, *p* < 0.001), (2) being victim of violence—other than sexual (χ^2^ = 12.701, *p* < 0.01), and (3) other major upheaval that may have shaped life or personality significantly (χ^2^ = 30.920, *p* < 0.001).

The differences in these domains of child trauma and total results were further checked by performing several Mann–Whitney *U*-tests. It showed that offenders, compared to healthy controls, had greater exposure to all of the three domains of childhood trauma (*U* = 396.500, 436.500, and 376.000, respectively; all significant at *p* < 0.001). Similar results were obtained when non-offenders were compared with healthy controls (*U* = 1223.000, 1535.000, and 999.000, respectively; all *p* < 0.001). Hence, non-offenders reported greater exposure to childhood trauma, compared to healthy controls. The differences between offenders and non-offenders were not statistically significant.

Confiding about childhood traumatic events was measured on a 7-point Likert scale (1–7), where higher scores indicated greater confiding. Comparisons in confiding between the two groups are also performed by using the Kruskal–Wallis test (Table 3), where higher mean ranks indicate higher confiding about different traumatic experiences during childhood.

**Table 3:** Results of Kruskal–Wallis test: differences between offenders, non-offenders, and healthy controls in confiding about childhood trauma.

**Confiding about traumatic experiences during childhood**	**Mean ranks**			**χ^2^**	***p***
	**Offenders (PSCH-V)**	**Non-offenders (Non-V-PSCH)**	**Healthy controls (HC)**		
1. Death of a very close friend or family member	32.92	38.03	35.96	0.536	0.765
2. Major upheaval between the parents (divorce, separation...)	24.38	24.74	28.00	0.688	0.709
3. Traumatic sexual experience (raped, molested...)	1.50	1.50	–	0	1
4. Victim of violence (child abuse, magged or assaulted, other than sexual)	19.05	19.34	21.54	0.421	0.810
5. Extremely ill or injured	11.60	14.22	12.64	0.526	0.769
6. Any other major upheaval that may have shaped life or personality significantly	18.79	29.83	28.23	5.630	0.060

*It was not possible to perform Kruskal—Wallis test with total scores due to large number of missing values*.

As can be seen from Table 3, the differences in confiding about childhood trauma between the three groups were not statistically significant in all cases. However, the chi-square test for the differences in the last domain of childhood trauma (other major upheaval that may have shaped life or personality significantly) was approaching borderline statistical significance (χ^2^ = 5.630, *p* = 0.060) with the lowest confiding (18.79) obtained for offenders with mental illness.

Table 4 shows that the differences in total exposure to childhood trauma was statistically non-significant between offenders who reported family history of alcoholism and those who did not. Similar results were obtained in the group of non-offenders. On the other hand, healthy controls who had a positive family history of alcoholism reported greater total exposure to childhood trauma (Mean rank = 46.72) compared to those without a family history of alcoholism (Mean rank = 27.36). The difference between mean ranks was statistically significant (Mann–Whitney *U* = 298.500, *p* < 0.001).

**Table 4:** Results of Mann–Whitney *U*-test: differences in traumatic experiences during childhood in relation to family history of alcohol abuse of parents.

**Groups**	**Family history of alcohol abuse**	**Total exposure to childhood trauma (mean ranks)**	***U***	***p***
Offenders (PSCH-V)	Yes	10.42	11.000	0.793
	No	12.00		
Non-offenders (Non-V-PSCH)	Yes	26.08	185.500	0.931
	No	25.61		
Healthy controls (HC)	Yes	46.72	298.500	<0.001
	No	27.36		

## Discussion

As expected, the highest total childhood trauma score was found in the PSCH-V sample. Statistically significant differences were obtained for total scores, as well as for (1) major upheaval between the parents, (2) being a victim of violence—other than sexual, and (3) other major upheaval that may have shaped life or personality significantly.

Results showed that the PSCH-V group, compared to the HC group, had greater exposure to all of the three domains of childhood trauma. Similar results were obtained when Non V-PSCH were compared with the HC group.

The differences between PSCH-V and Non-V-PSCH were not statistically significant. Although no statistically significant differences were found, the total CTQ score in the PSCH-V group was higher (103.5), in comparison to the Non-V-PSCH group (83.89). This suggests the presence of a larger number of traumatic events in childhood. This result is in accordance to the previous studies that have shown that the presence of a large number of traumatic events in childhood in one person affects the frequency of violent behaviors in the community (1, 2).

These results confirm that the intensity and presence of a greater number of traumas in childhood increase the likelihood of violent behavior in adulthood, but also the occurrence of psychotic illnesses (14).

A statistically significant difference was found between CTQ scores between both groups of patients with psychosis and schizophrenia (PSCH-V and Non-V-PSCH) compared to the HC group.

The differences in confiding about childhood trauma between the three groups were not statistically significant in all cases. However, the chi-squared test for the differences in the last domain of childhood trauma (other major upheaval that may have shaped life or personality significantly) was approaching borderline statistical significance (χ^2^ = 5.630, *p* = 0.060) with the lowest confiding (18.79) obtained for PSCH-V with mental illness.

The variable “other major upheaval that may have shaped life or personality significantly” was present predominantly in the PSCH-V group who cited the trauma and abuse they suffered due to their parents' alcohol abuse in response to this question.

The consequences of childhood abuse can be reflected in the occurrence of homicidal or suicidal behavior, specifically in traditional settings, where men have the role of the main head of the household and experience less freedom to confide in others. These results are consistent with Bowlby's attachment theory. Bowlby (15) demonstrated that the ultimate goal of attachment behavior is to discover an integrated system with which to organize the self, in order to grow, learn and develop.

The manner in which the infant begins to learn and develop depends upon the nature of the attachment figure. If the child has no secure base to organize itself around (as in a disorganized attachment pattern), this can lead to a disintegrated and fragmented sense of self and others. A disorganized attachment pattern results from a situation in which inconsistent reactions of parent figures and experiences of fear leave a child with a lack of safety and no understanding of what is secure (16).

Traumatic experiences can contribute to neurodevelopmental difficulties with regulating emotion and disrupted attachments can contribute to a fragmented understanding of the “self” and the external world. Dissociation resulting from trauma can also cause confusion, concentration problems, disorientation, and disorganization. Emotional regulation can be seriously altered following trauma and this can have a marked impact on reality testing—this can become a factor in the development of psychotic symptoms (17).

Excessive traumatic stimulation sensitizes neuronal circuits, in a sense enhancing natural in-built stress responses (fight/flight/freeze). This hypersensitivity to threat can become “hard-wired” and can lead to hyperarousal symptoms and a cognitive bias toward interpreting something safe as being threatening. With all the evidence presented above, the manner in which paranoid delusions can arise in an individual whose threat system is repeatedly and erroneously triggered becomes more understandable within the context of traumatic experiences and dissociative processes (17).

The results of this study show that there are statistically significant differences between childhood traumatic events experienced by patients with psychosis and schizophrenia (PSCH-V and Non-V-PSCH) compared to HC, which can be explained by the fact that childhood traumatic events have increased the risk of developing psychotic disorders and schizophrenia in adulthood compared with the general population (3, 18).

In this paper, among the significant clinical characteristics of PSCH-V patients, parental alcohol abuse showed to be an important variable. We have identified 60% of parents who were abusing alcohol, while in the Non-V-PSCH group, parental alcohol abuse was present in 35% of cases. In HC, the presence of parental alcohol abuse was found in 5.5% of cases. The V-PSCH and Non-V-PSCH patients claimed that their fathers manifested extremely aggressive behavior under the influence of alcohol. According to their reports, alcohol abusing fathers were prone to physical punishments, verbal violence such as using insults and threats, as well as aggressive behavior toward the other parent. Patients cited this as a significant stress factor in childhood, because poor parental relationships and their physical and emotional conflicts induced feelings of insecurity, anxiety, guilt, and perception of lacking social support.

The differences in total exposure to childhood trauma were statistically non-significant between PSCH-V and Non-V-PSCH patients who reported a family history of alcohol abuse and those who did not. On the other hand, participants from the HC group who had a positive family history of alcohol abuse reported greater total exposure to childhood trauma compared to those without a family history of alcohol abuse. Due to the larger HC sample, a statistically significant difference was demonstrated. Although the study included the largest number of patients treated in the Court Department, such a small sample may pose a problem for statistical processing, and can lead to type II error. We assume that if the sample was larger we would get a similar result for the PSCH-V and Non-V-PSCH groups as for the HC group. For this reason, we recommend further research on a larger sample.

According to studies that examined the impact of childhood abuse in the general population, there is an increased risk of violent and criminal behavior later in adulthood in children who have been victims of violence in childhood. The conclusions of some studies indicate that this finding is in accordance with the model of transgenerational transmission of violence (1, 2, 19). Research has shown that victims of childhood abuse also have an increased risk of developing mental disorders that include personality disorder, schizophrenia, psychosis, and substance abuse. These are predominantly present in forensic mental health inpatients (3, 4).

The results of our study show that in both PSCH-V and Non-V-PSCH groups, the presence of the mental illness of the parents was statistically significantly higher than in HC. Furthermore, results show that there is no statistically significant difference between the PSCH-V and Non-V-PSCH group in the family inheritance of mental illness. Presence of parents' alcohol abuse in the PSCH-V group, along with other forms of abuse and neglect in childhood, may be a factor relevant to violent behavior and may explain the difference in violence between the PSCH-V group and the other two groups, through the of model intergeneration transmission of violence. These results indicate the need for further research on this topic.

Statistically significant differences were obtained for total scores, as well as for (1) major upheaval between the parents, (2) being a victim of violence—other than sexual, and (3) other major upheaval that may have shaped life or personality significantly. Results showed that the PSCH-V group, compared to the HC group, had greater exposure to all of the three domains of childhood trauma. Similar results were obtained when Non V-PSCH were compared with the HC group. Hence, Non V-PSCH reported greater exposure to childhood trauma, when compared to HC. The differences between PSCH-V and Non V-PSCH were not statistically significant. Although no statistically significant differences were found, the total CTQ score in the PSCH-V group was higher (103.5), in comparison to the Non V-PSCH group (83.89), which suggests the presence of a larger number of traumatic events (cumulative polyvictimization) in childhood. This result is in accordance to the previous studies that have shown that the presence of a large number of traumatic events in childhood in one person affects the frequency of violent behaviors in the community (1, 2).

## Limitations

One limitation of the study is the subjective testimony of patients regarding their responses on the CTQ scale. Memories of traumatic childhood events can be altered, repressed, forgotten, and in some cases deliberately concealed. Also, there is a possibility that some of the patients underestimated or felt ashamed due to some traumatic events, so they did not report them. In the same manner, they could have overestimated them, especially in the HC group. Nevertheless, studies to date have shown that the CTQ questionnaire gives reliable results.

Another limitation was the small number of patients in the PSCH-V group. Although the study included the largest number of patients treated in the Court Department, such a small sample may pose a problem for statistical processing, and can lead to type II error. Therefore, replication of this study with a larger sample is required.

Limitation is also the impossibility to examine the transmission of violence through several generations, and this paper refers to the transmission from one generation to the next, given that the examination was done with only one generation of patients.

## Conclusion

Statistically significant differences were obtained for total scores, as well as for: major upheaval between the parents, being victim of violence—other than sexual, and the major upheaval that may have shaped life or personality significantly. For both groups of patients with the psychotic disorder from the spectrum of psychosis and schizophrenia (PSCH-V and Non V-PSCH) statistically, significant differences in these four domains were found, relative to HC. In the PSCH-V group, a significant presence of parental alcohol abuse was found (60%). Along with other forms of abuse and neglect in childhood, parental alcohol abuse can be a trigger for violent behavior, and an explanation for the higher levels of violence found in the PSCH-V group in comparison to the other two groups. Plausible mechanism for violent behavior later in adulthood can be the intergenerational transmission of violence. Although no statistically significant differences were found, the overall CTQ score in the PSCH-V group was higher (103.5) in comparison to the Non V-PSCH group (83.89), suggesting the presence of a greater number of traumatic events (cumulative polyvictimization) in childhood. Parents who are abusing alcohol have a reduced ability to provide a safe environment for their children and to respond adequately to children's physical and emotional needs. Families in which the parent is abusing alcohol are more likely to experience verbal and physical conflicts between their children and spouses, and therefore manifest a reduced ability to provide their children with security, emotional and physical needs, as well as economic security. Prevention of alcohol abuse as well as all forms of abuse and neglect of children should be the focus of professionals in the field of mental health, health policies, and society as a whole in order to prevent violence.

## Data Availability Statement

The original contributions presented in the study are included in the article/Supplementary Material, further inquiries can be directed to the corresponding author/s.

## Ethics Statement

The studies involving human participants were reviewed and approved by Ethics Committee of Medical Faculty University of Montenegro. The patients/participants provided their written informed consent to participate in this study.

## Author Contributions

MP has taken part in the study design, inclusion of patients and control group participants, and has been writing and editing the paper. LI has assisted in selection of the PSCH-V group and took active role in writing and editing of the paper, as well as in the study design. MP and LI interpreted statistical analyses. Both authors contributed to the article and approved the submitted version.

## Funding

This work was supported by Ph.D. Scholarship of the Ministry of Education, Science, Culture, and Sports of Montenegro.

## Conflict of Interest

The authors declare that the research was conducted in the absence of any commercial or financial relationships that could be construed as a potential conflict of interest.

## Publisher's Note

All claims expressed in this article are solely those of the authors and do not necessarily represent those of their affiliated organizations, or those of the publisher, the editors and the reviewers. Any product that may be evaluated in this article, or claim that may be made by its manufacturer, is not guaranteed or endorsed by the publisher.
