# Supplementary material for: Intergeneration Transmission of Violence in Forensic Patients With a Diagnosis of Schizophrenia and Psychosis: Was Parental Alcoholic Abuse a Significant Factor?
Source: Front Psychiatry. 2021 Dec 2;12:765279. doi: 10.3389/fpsyt.2021.765279 (PMC8675210; doi:10.3389/fpsyt.2021.765279)
Supplement: Supplementary file 1 [file Data_Sheet_1.zip › Table 4.DOCX]

| Groups | Family history  of alcoholism | Total exposure to  childhood trauma  (Mean Ranks) | | | U | | p | |
| --- | --- | --- | --- | --- | --- | --- | --- | --- |
| Offenders  (PSCH-V) | Yes  No | 10.42  12.00 | | 11.000 | | | .793 | |
| Non-offenders  (Non-V-PSCH) | Yes  No | 26.08  25.61 | 185.500 | | | .931 | |  |
| Healthy controls  (HC) | Yes  No | 46.72  27.36 | 298.500 | | | <.001 | |  |

*Table 4. Results of Mann-Whitney U test: Differences in confiding about traumatic experiences during childhood in relation to family history of alcoholism of parents*

Table 4 shows that the differences in total exposure to childhood trauma was statistically non-significant between offenders who reported family history of alcoholism and those who did not. Similar result was obtained in the group of non-offenders. On the other hand, healthy controls who had a positive family history of alcoholism reported greater total exposure to childhood trauma (Mean rank = 46.72) compared to those without family history of alcoholism (Mean rank = 27.36). The difference between mean ranks was statistically significant (Mann-Whitney U = 298.500, p < .001).
