# Supplementary material for: Intergeneration Transmission of Violence in Forensic Patients With a Diagnosis of Schizophrenia and Psychosis: Was Parental Alcoholic Abuse a Significant Factor?
Source: Front Psychiatry. 2021 Dec 2;12:765279. doi: 10.3389/fpsyt.2021.765279 (PMC8675210; doi:10.3389/fpsyt.2021.765279)
Supplement: Supplementary file 1 [file Data_Sheet_1.zip › Table 5.DOCX]

Descriptive values (frequencies) regarding the experiences of various domains of childhood trauma are shown in Figure 1.


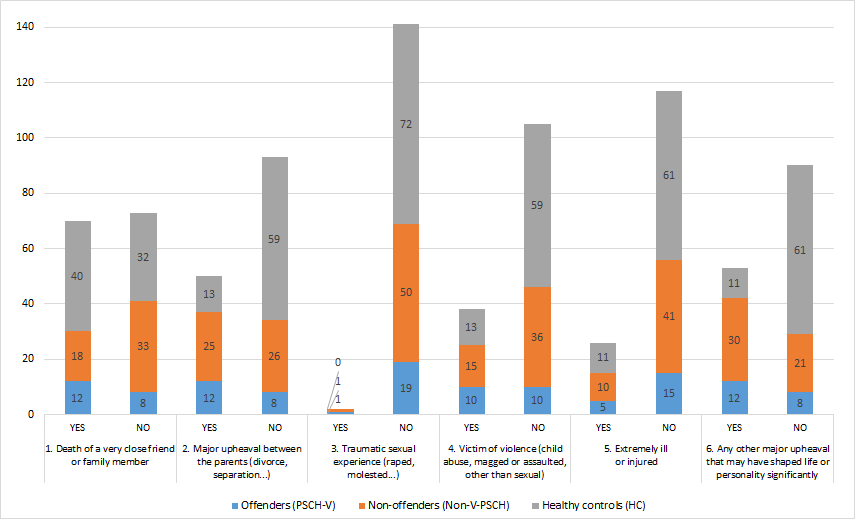


*Figure 1. Frequencies of participants who have or have not experienced each domain of childhood trauma*
